# Applying the Learning Health System framework to overcome barriers in antimicrobial decision support: lessons from the Guidance redesign

**DOI:** 10.1017/ash.2025.10222

**Published:** 2025-11-17

**Authors:** Jenna Maleki, Lisa Hall, Karin Thursky

**Affiliations:** 1 Royal Melbourne Hospital Guidance Group, Melbourne Health, Melbourne, VIC, Australia; 2 National Centre for Antimicrobial Stewardship, Department of Infectious Diseases, University of Melbourne, Melbourne, VIC, Australia; 3 School of Public Health, The University of Queensland, Brisbane, QLD, Australia

## To the Editor,

We read with great interest the narrative review by Bosetti et al.,^1^ which highlights that despite their theoretical benefits, clinical decision support systems (CDSS) for antimicrobial prescribing often fail to achieve sustained clinical impact because of poor workflow integration, low user engagement, inadequate user-centered design, and limited evaluation within rigorous research frameworks. The authors’ discussion of implementation challenges, particularly those illustrated by the COMPASS trial,^2^ resonates strongly with our experience at the Royal Melbourne Hospital (RMH) during the redevelopment of *Guidance*, a rule-based antimicrobial CDSS supporting a systemwide approach to antimicrobial stewardship (AMS) across Australian hospitals.

When the RMH Guidance Group embarked on the redesign of *Guidance MS* to the new *Guidance* platform, we faced the very barriers Bosetti et al describe. Initially introduced in 2005, *Guidance MS* functioned primarily as a preprescription decision support and approvals program. However, over time we recognized that maintaining clinical impact required more than robust rulesets, it demanded better workflow integration, attention to user experience, and continuous evaluation.

To address these limitations, *Guidance* was redeveloped using a Learning Health System (LHS) framework.^3^ This approach enabled iterative co-design, seamless integration with hospital systems, and real-time learning from user feedback. This is precisely the multifaceted strategy advocated by Bosetti et al for effective and sustainable CDSS deployment.

A multidisciplinary LHS community was established, comprising infectious diseases physicians, pharmacists, nurses, software engineers, and executive sponsors. Central to the redevelopment was a clinician-led core team supported by a dedicated UI/UX designer, ensuring that usability and workflow fit were considered from the outset. User feedback was systematically captured through community forums, focus groups, and support logs and then thematically analyzed to inform design priorities. These insights directly shaped enhancements such as:

- interoperability with electronic medical records (EMRs), patient administration systems, and a SNOMED-CT–coded indication list;
- adherence to Australia’s Therapeutic Goods Administration (TGA) *Essential Principles for Software as a Medical Device* and ISO 9001/62366 standards, underpinned by a robust Quality Management System;
- interactive dashboards providing real-time feedback on antimicrobial prescribing patterns and stewardship activity.

Design Thinking principles underpinned a user-centered redesign, ensuring that each feature addressed the practical needs of clinicians and reduced unnecessary cognitive and procedural burden. Human factors considerations were systematically embedded across all components, focusing on clarity, efficiency, and safety. User personas were developed to represent key clinician groups and workflows, allowing the design team to empathize with real-world use cases and align functionality with day-to-day clinical practice.

Using Agile sprints, iterative usability testing, and continuous stakeholder feedback, we optimized both system performance and clinician experience. Each interface component underwent refinement to ensure intuitive use in time-critical settings, directly addressing the workflow integration and perceived time-burden issues identified in the COMPASS trial.^2^ One-way EMR integration was achieved at rollout, and two-way “write-back” functionality is now under active development through our Clinical Decision Support Hooks project. Upcoming enhancements include a redesigned mobile interface and improved content-authoring workflows to support local adaptation.

Importantly, the LHS framework enabled rapid iteration during implementation, a feature that proved critical as new functionality was requested in real time. This dynamic adaptation helped maintain user trust and avoided the disengagement that often undermines CDSS adoption. Our experience reinforces Bosetti et al.’s conclusion that ongoing socio-technical evaluation, not static implementation, is essential to sustain clinical impact.

Aligned with the authors’ call for rigorous evaluation, we have secured ethics approval for a multi-site evaluation across several Australian hospitals. This evaluation will extend beyond traditional process measures (antimicrobial appropriateness and consumption) to assess clinical outcomes (mortality, length of stay), economic metrics, and user perceptions of usability and usefulness. Continuous feedback cycles within the LHS will enable design refinements, thereby closing the “evaluation–implementation” gap that Bosetti et al identify as a persistent weakness in the field.

Our experience suggests that many of the challenges detailed in the review, such as low adoption, poor workflow fit, and lack of evaluation frameworks, can be systematically addressed through an LHS-based, human-centered approach that embeds evaluation, regulation, and design within a single iterative ecosystem. By grounding development in human factors, regulatory compliance, and continuous learning, *Guidance* provides a scalable and sustainable model for CDSS-enabled AMS.

In summary, we concur with Bosetti et al that CDSS alone will not change prescribing practice unless implementation science and user-centered design principles are integral to development. The LHS framework offers a practical methodology to operationalize these principles, ensuring that CDSS evolve in partnership with their users, generate real-world evidence of effectiveness, and ultimately achieve the sustained behavioral change necessary for AMS success.

Sincerely,

Jenna Maleki, Lisa Hall, and Karin Thursky
